# Bidirectional Longitudinal Relationships Between Homebound Status and Falls Among Older Adults

**DOI:** 10.1093/geroni/igaa057.856

**Published:** 2020-12-16

**Authors:** Minhui Liu, Yuxiao Li, Xiaocao Sun, Christina Miyawaki, Tianxue Hou, Siyuan Tang, Sarah Szanton

**Affiliations:** 1 Central South University, Baltimore, Maryland, United States; 2 Central South University, Changsha, Hunan, China; 3 Central South University, Changsha City, Hunan, China; 4 University of Houston, Houston, Texas, United States; 5 Johns Hopkins University, Baltimore, Maryland, United States

## Abstract

Research has shown an association between homebound status and falls among older adults. However, this association was primarily drawn from cross-sectional studies. Using the National Health and Aging Trends Study, we examined 1) whether prior-wave falls predicted homebound status in a later wave in 2,916 non-homebound participants in Wave 1 and 2) whether prior-wave homebound status predicted falls in 2,512 participants with no falls in Wave 1. Homebound status (non-homebound and homebound) was determined by the frequency, difficulty, and needing help of outdoor mobility. Falls were ascertained by asking participants whether they had a fall in the last year. Generalized estimation equation models were used to examine their bidirectional association, adjusting for demographics, health-related, and behavioral factors. Participants who had fallen in later waves were more likely to be older non-Hispanic black, comorbid, and have more pain, depression, disabilities, worse health status vision impairment, and low physical activities. Participants who were homebound in later waves tended to older, female, non-Hispanic black, less-educated, living alone or with others only, comorbid, obese, and have more pain, depression, disabilities, worse health status, more hospitalizations, vision and hearing problems, and low physical activities. Previous falls significantly predicted later homebound status (adjusted odds ratio [OR]: 1.28, 95% CI: 1.09-1.50). Prior wave homebound status also significantly contributed to falls in the next year (adjusted OR: 1.28, 95% CI: 1.12-1.46). The bidirectional longitudinal association between homebound status and falls suggests a vicious circle between them. Fall prevention programs should particularly target homebound older adults for falls reduction.

